# A case of small intestinal endometrioid adenocarcinoma

**DOI:** 10.1186/s40792-016-0226-6

**Published:** 2016-09-14

**Authors:** Yusuke Ogi, Tomohiro Yamaguchi, Yusuke Kinugasa, Akio Shiomi, Hiroyasu Kagawa, Yushi Yamakawa, Masakatsu Numata, Akinobu Furutani, Masakazu Abe

**Affiliations:** 1 Division of Colon and Rectal Surgery, Shizuoka Cancer Center Hospital, 1007 Shimonagakubo, Nagaizumi-cho, Sunto-gun, Shizuoka 411-8777 Japan; 2 Division of Gynecology, Shizuoka Cancer Center Hospital, 1007 Shimonagakubo, Nagaizumi-cho, Sunto-gun, Shizuoka 411-8777 Japan

**Keywords:** Endometriosis, Malignant transformation, Small intestine, Magnetic resonance imaging

## Abstract

Endometriosis generally occurs in the ovary. Intestinal endometriosis is rare. About 1 % of all endometriosis cases become malignant. Malignant transformation of small intestinal endometriosis is very rare. A 55-year-old woman who underwent total abdominal hysterectomy and bilateral salpingo-oophorectomy and omentectomy for endometriosis 7 years ago presented to her primary care doctor with melena. A tumor was detected in the right lower abdomen by ultrasonography. The doctor referred her to our hospital. Computed tomography demonstrated a lobulated tumor ventral to the right common iliac vessels. Magnetic resonance imaging demonstrated that the tumor had heterogeneous intensity on T2-weighted images. Several small cysts with high intensity were observed caudal to the tumor on T2-weighted images. We performed partial small intestinal resection for the lesion. The tumor was diagnosed as endometrioid adenocarcinoma of the small intestine. She has been relapse-free for 5 years after surgery. Only three cases of malignant transformation of small intestinal endometriosis have been reported previously. It is very rare for long-term survival to be obtained with surgery alone, as in our case. This case report highlights the imaging findings for malignant transformation of intestinal endometriosis.

## Background

Endometriosis generally occurs in the ovary. It rarely involves the intestinal tract. Almost all reported cases of intestinal endometriosis involve the sigmoid colon and rectum [1]. There have been relatively few reports of endometriosis in the small intestine. Malignant transformation occurs in approximately 1 % of all endometriosis cases [2]. We report a very rare case of endometrioid adenocarcinoma, which is endometriosis with malignant transformation, in the small intestine.

## Case presentation

A 55-year-old woman presented to her primary care doctor with melena. A tumor in the right lower abdomen was detected by ultrasonography. The doctor referred her to our hospital for further examination and treatment. She underwent total abdominal hysterectomy and bilateral salpingo-oophorectomy and omentectomy for endometriosis 7 years ago.

**Fig. 1:**
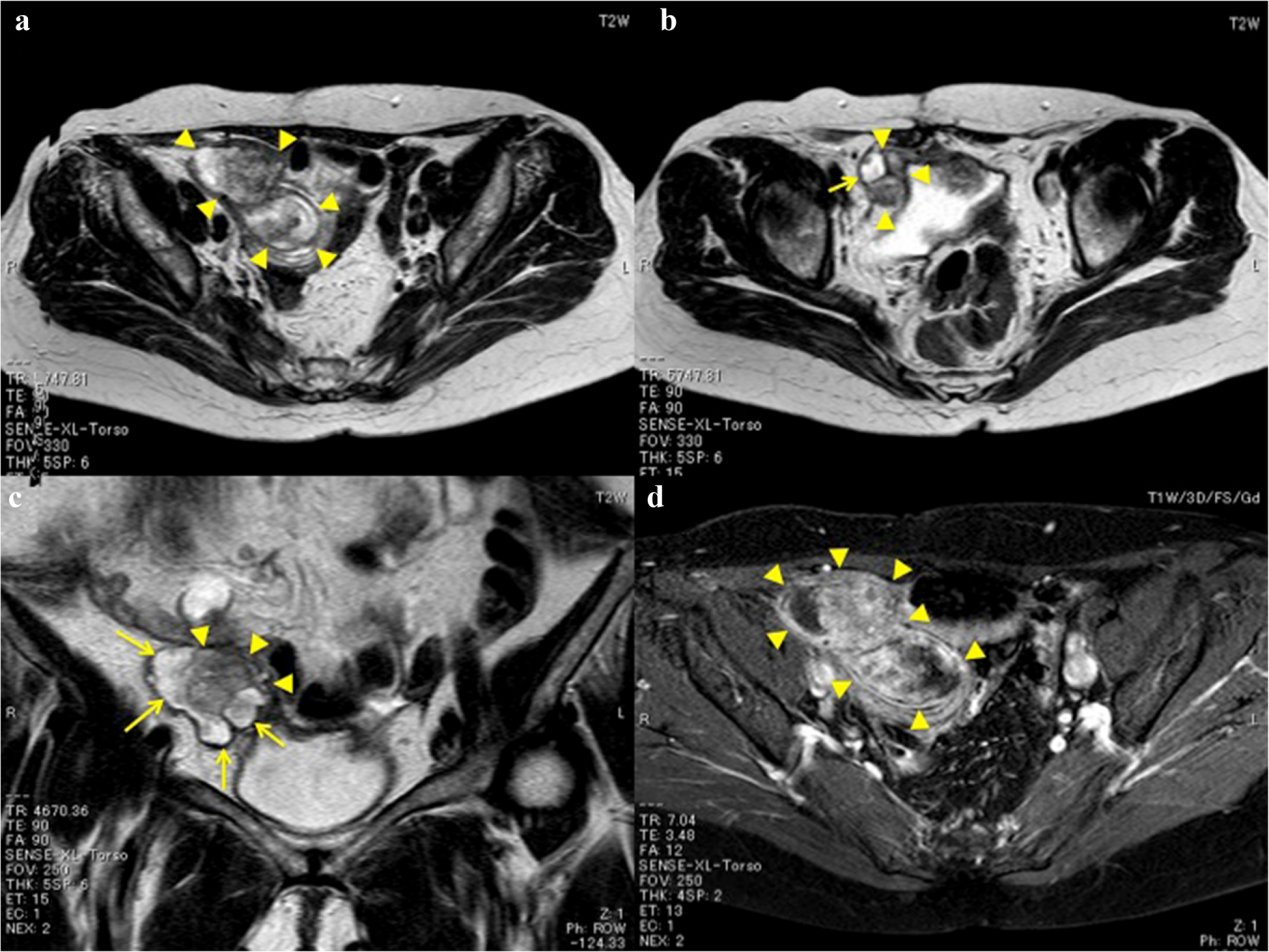
MR imaging. **a** T2-weighted imaging. A lobulated tumor was observed ventral to the right common iliac artery. The tumor had heterogeneous intensity. **b**, **c** T2-weighted imaging. Some high-intensity cystic lesions (*arrow*) were detected around the tumor. **d** T1-weighted imaging. The mass had heterogeneous enhancement (*arrowheads* indicate the outline of the tumor)

On physical examination, a tumor was slightly palpable in the right lower abdomen. Computed tomography of the abdomen demonstrated a 6.5 × 4 cm lobulated tumor ventral to the right common iliac vessels. Enlarged lymph nodes were observed in the area of the ileocolic artery. She also underwent a contrast-enhanced magnetic resonance (MR) imaging examination. The tumor had intermediate signal intensity, similar to that of muscle, on T1-weighted images and heterogeneous intensity on T2-weighted images. On contrast-enhanced MR images with gadolinium, the mass was enhanced heterogeneously. Several small cysts that were hypointense on T1-weighted images and hyperintense on T2-weighted images were observed caudal to the tumor (Fig. 1).

Blood tests revealed anemia, with a hemoglobin concentration of 8.5 g/dL. Serum concentrations of carcinoembryonic antigen and carbohydrate antigen 125 were 1.7 ng/mL and 14 U/mL, respectively. Endoscopic examination did not detect any tumors or sites of bleeding in the colon.

**Fig. 2:**
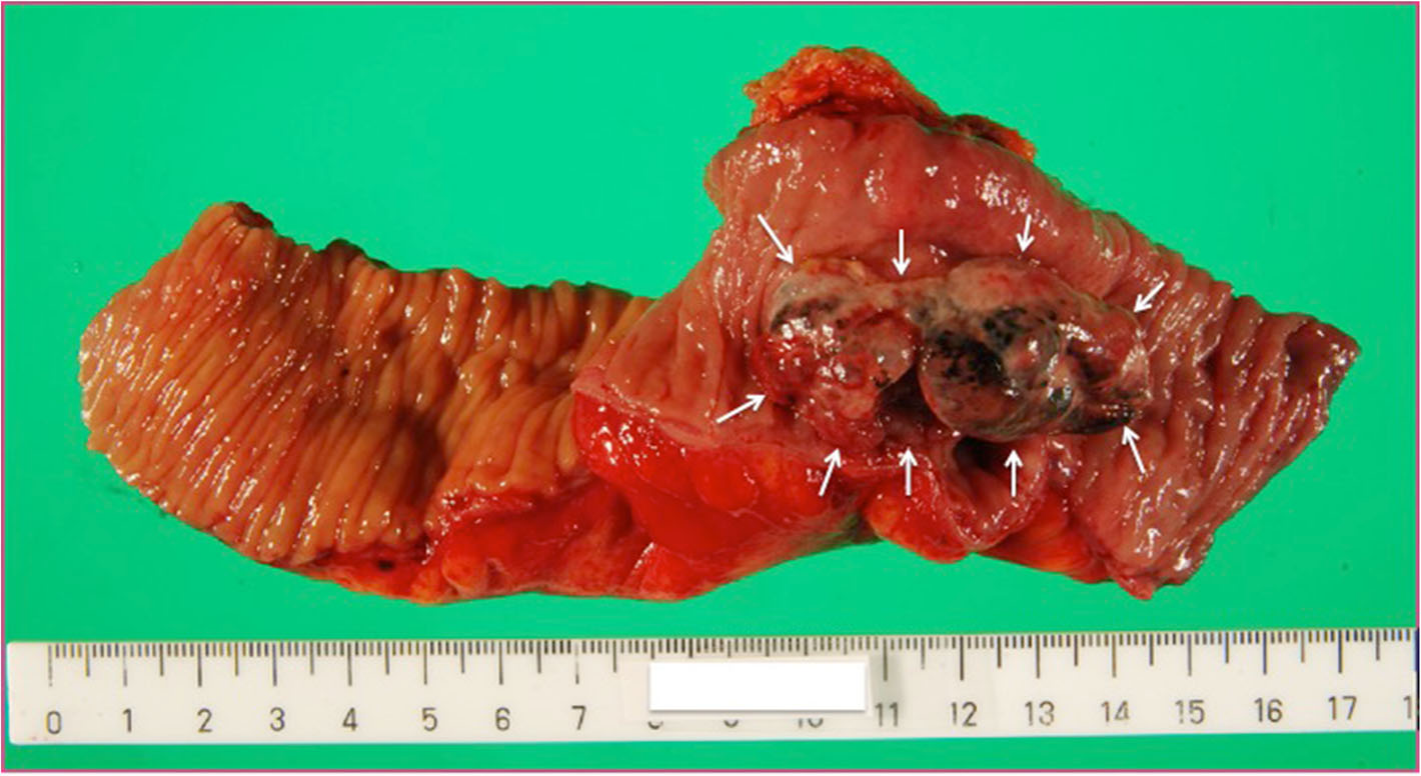
Macroscopic findings. The tumor (*arrows*) invaded the intestinal mucosa

We suspected primary small intestinal cancer, a retroperitoneal tumor with invasion to the small bowel, or endometriosis with malignant transformation. Surgery was performed. Intraoperatively, we observed that the tumor was located ventral to the right common iliac vessels. Because the tumor invaded the ileum 35 cm proximal to the end of the ileum, we performed partial small intestinal resection (Fig. 2).

**Fig. 3:**
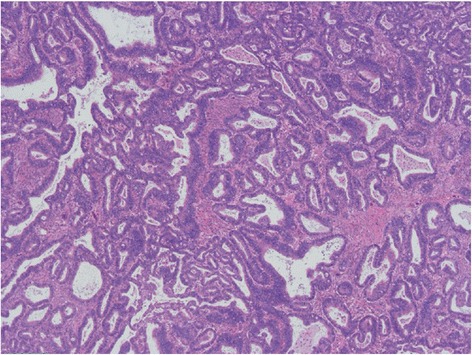
Microscopic findings. Sixteen tumor cells had nuclear atypia and irregular glandular structures (hematoxylin-eosin 17 stain, ×40)

Histologically, the tumor penetrated to the mucosa of the small intestine. The tumor consisted of two types of stromal tissue (endometriosis type and ovarian type) and atypical endometrioid glandular epithelium. The atypical cells were immunopositive for cytokeratin 7 but were negative for cytokeratin 20. The histopathological diagnosis was endometrioid adenocarcinoma (Fig. 3). The stromal tissues in the wall of the small intestine surrounded the adenocarcinoma component. The endometrioid adenocarcinoma was localized in the intestinal wall. Small intestinal endometriosis was considered the site of malignant transformation. There was no lymph node metastasis. Postoperative chemotherapy was not performed. She has been relapse-free for 5 years after surgery.

### Discussion

The clinical course of this patient illustrates two important clinical points. Malignant transformation of endometriosis can occur in the small intestine. MR imaging is useful in the diagnostic evaluation of malignant endometriosis in the small intestine.

First, malignant transformation of endometriosis can occur in the small intestine. The ovary is the site of 80 % of all endometriosis cases. Abdominal endometriosis accounts for 5.3 to 12 % of endometriosis [3]. Intestinal endometriosis mainly occurs in the sigmoid colon and rectum, and these two sites account for 77 % of abdominal endometriosis [1]. About 1 % of all endometriosis cases become malignant [2]. Only three cases of malignant endometriosis in the small intestine have been reported (Table 1) [4–6]. It is very rare for long-term survival to be obtained with surgery alone, as in our case.

**Table 1:** Reports of endometriosis with malignant transformation in the small intestine

Case	Author	Year	Age	Past surgical history	Histology	Postoperative therapy	Survival	Reference
1	Ferraro et al.	1956	44	1. Left partial oophorectomy for endometriosis	Endometriotic sarcoma	Radiation therapy	Died of disease 4 months after surgery	[4]
				2. Right oophorectomy for corpus luteum cyst				
				3. Supravaginal hysterectomy for fibromyoma				
				4. Removal of a right intraligamentous chocolate cyst with endometriosis				
				5. Colon resection for pelvic endometrioma				
2	Baiocchii et al.	1990	38	TAH and BSO for endometriosis	Endometrioid stromal sarcoma	Chemotherapy	No contributions	[5]
3	Makihara et al.	2015	25	None	Endometrioid adenocarcinoma	None	Disease-free for 10 months	[6]
4	Present case	2016	55	TAH and BSO and omentectomy for endometriosis	Endometrioid adenocarcinoma	None	Disease-free for 5 years	–

*TAH* total abdominal hysterectomy, *BSO* bilateral salpingo-oophorectomy

Second, MR imaging is useful for diagnosing malignant endometriosis in the small intestine. Endometriosis in the rectum is often assessed with MR imaging, with very high diagnostic accuracy. Compared to rectal endometriosis, the diagnostic accuracy of intestinal endometriosis with MR imaging is lower [7]. Endometriosis in the acute phase is characterized by high signal intensity on both T1- and T2-weighted images, which is suggestive of the presence of subacute hemorrhage. During the chronic phase, shading, which is defined as a centrally or peripherally located low-intensity area in a hyperintense cyst, can be observed on T2-weighted images [8]. If solid nodules exist in a cyst, there is a possibility of malignant transformation. Contrast-enhanced MR imaging with gadolinium is considered when solid components of endometriosis are suspected to be malignant. In the study of mural nodules within cysts, malignant endometriosis is characterized by the lack of shading on T2-weighted images, high signal intensity on diffusion-weighted images, and heterogeneous enhancement on MR images with contrast [9]. In our case, the tumor had surrounding small cysts with low intensity on T1-weighted images, heterogeneous intensity on T2-weighted images, and heterogeneous enhancement on T1-weighted images with contrast. Small cysts are considered to reflect endometriosis, while heterogeneous enhancement on T1-weighted images reflects malignant transformation. In patients with a history of endometriosis, when a tumor with cysts is detected, we need to consider the possibility of malignant transformation arising from intestinal endometriosis. Contrast-enhanced MR imaging may be useful in the diagnostic evaluation of malignant endometriosis in the small intestine.

Surgical resection is the first-choice treatment for malignant endometriosis. Lymph node metastasis and peritoneal dissemination have been reported to be poor prognostic factors [10, 11]. Patients with either factor may benefit from postoperative chemotherapy. In the case of endometrioid adenocarcinoma of the ovary, the recommended postoperative chemotherapy regimen is carboplatin plus paclitaxel [12, 13]. If the tumor is localized and well differentiated and there is no lymph node metastasis, postoperative chemotherapy may be omitted in ovarian cancer. However, the evidence base for postoperative chemotherapy to treat intestinal malignant endometriosis has not been fully established because of the small number of cases. In the present case, endometrioid adenocarcinoma was localized to the intestinal wall, resection margins were negative, and complete resection was performed. There was no lymph node metastasis and histological examination showed that the tumor was well differentiated. We consulted with the patient and decided not to perform postoperative chemotherapy.

## Conclusions

Malignant endometriosis can arise in the small intestine and MR imaging is useful for diagnosing endometriosis with malignant transformation. Although there is a need to accumulate more cases, we propose that contrast-enhanced MR imaging is useful for detecting malignant transformation of intestinal endometriosis.

## Abbreviations

BSO: bilateral salpingo-oophorectomy
MR: magnetic resonance
TAH: total abdominal hysterectomy
